# The clinician-scientist track: an approach addressing Australia’s need for a pathway to train its future clinical academic workforce

**DOI:** 10.1186/s12909-018-1337-5

**Published:** 2018-10-03

**Authors:** Diann S Eley

**Affiliations:** Office of Medical Education, Faculty of Medicine, The University of Queensland, 288 Herston Road, Brisbane, QLD 4006 Australia

**Keywords:** Clinician-scientist, Medical student, MD-PhD, MD-masters, Master’s degree, PhD degree, Research training, Clinical academic, Physician-scientist, MPhil

## Abstract

**Background:**

Clinician-scientist training represents the epitome of preparation for biomedical scientific discovery. The significance of, and need for, clinician-scientists is universally recognised as essential to progress medical research across what is regarded as the ‘translational gap’. Despite a rich history of cutting-edge biomedical research, Australia has no infrastructure or career pathway for training clinician-scientists.

**Discussion:**

The Clinician-scientist Track (CST) was developed to address this concern at the University of Queensland. The CST concept began in 2010 with the Concurrent MD-Masters that allowed students to undertake a research Masters concurrently with their medical program. The rationale was to offer an attractive and realistic option to recruit our highest performing students into a research higher degree, with the underlying aim of encouraging those most capable, to transfer to the MD-PhD. The Concurrent MD-Masters was immediately popular and remains so. Over 8 years, enrolments rose seven-fold (60 MD-Masters, 36 MD-PhDs). The transfer rate from MD-Masters to MD-PhD is 28% supporting our original aim.

**Conclusions:**

Many challenges remain for the future of the program. These challenges are underpinned by a culture that values clinician-scientists as crucial to ensuring that high quality health and medical research is undertaken and translated to patient care, but lags behind in establishing an infrastructure to develop and maintain a new generation of this vital workforce. A future challenge is to develop a coordinated approach to a supported Australian MD-PhD pathway for our most talented and committed students beginning in the undergraduate Bachelor’s degree into the medical degree and throughout specialty training. Shared responsibility is necessary between institutions and stakeholders to support and nurture newly trained MD-PhDs into the post-graduate years. Flexibility across this medical training continuum that allows integration of both degrees will help ensure students make the most meaningful connections between the research and the medicine. What is paramount will be acknowledging the career expectations of an emerging cohort of medical students, in particular females, wishing to pursue research. Without these considerations we risk losing our next generation of potential clinician-scientists.

## Background

Not without its challenges, clinician-scientist training remains the epitome in preparation for biomedical research. Clinician-scientists are clinically trained health professionals who have undergone additional training in research, typically a PhD, and include research as a significant part of their professional career. The significance of, and need for, clinician-scientists is universally recognised as essential to progress medical research across what is regarded as the ‘translational gap’ [1]. This gap covers a broad range of research from clinical trials to community health. The ultimate goal is to implement research to enhance health care through sustainable improvements in patient outcomes. Despite this recognition, there continues to be a global decline in the numbers of clinician-scientists. In Australia there have been two summits devoted to this dilemma which emphasised the urgent task of training and reinvigorating its clinician-scientist workforce [2, 3].

In medicine, the MD-PhD is a dual-degree recognised worldwide, and often accepted as the main route to clinician–scientist training [4–6]. In the United States (US), the Medical Scientist Training Program (MSTP) [7] established in 1964, continues to provide the majority of support for MD-PhD training to students at select medical schools [8]. Canada has a similar MD-PhD model [9, 10]. However, MD-PhD programs are not the only approach to clinician-scientist training. In the UK, clinician-scientist training focuses on a continuum within the post-graduate training years. The Clinician Scientist Fellowship (CSF), as part of the Academy of Medical Sciences recruitment into academic medicine, allows protected time for post-doctoral research during speciality training [11, 12]. The structure of the MSTP and the CSF represent clear pathways to a combined career in medicine and scientific discovery. The notion of a continuum of training or ‘pipeline’ more accurately reflects the realities of the clinician-scientist training. While Milewicz et al. [13] describe this pipeline as ‘long and leaky’; it nevertheless represents a range of opportunities for students, trainees or fellows to negotiate their journey across years of arduous training.

### Australia’s context

In Australia, the importance of clinician-scientists and the quality of medical and research training is no less valued. According to the 2018 Times Higher Education World University Rankings, five of Australia’s 43 universities are listed in the world’s top 100 and three of its 18 medical schools in the world’s top 50 [14, 15]. Surprisingly, in Australia, there is no national approach to clinician-scientist training in either the undergraduate or postgraduate training years. There is no clear pathway for research higher degree training for medical students, junior doctors or registrars as they move through to their clinical fellowship [16–20].

Until 2015, most Australian medical schools offered a four-year MBBS degree (Bachelor of Medicine Bachelor of Surgery). Currently, all but seven of Australia’s 21 medical schools now offer the four-year graduate entry MD degree. Combining a research higher degree (RHD), comprising a research Masters or a PhD, with medical training is uncommon in Australia. For clarification*: a research Master’s degree in Australia is equivalent to the Master of Science (MSc) in the US. However, the degree is strictly research with no compulsory course work and is referred to as a Master of Philosophy or MPhil. This paper will refer to this RHD as a Masters*. Nevertheless, most Australian medical schools offer a means for eligible students to undertake a Masters or PhD with the MD, sometimes referred to as a dual-degree. This follows the traditional MD-PhD structure of intercalating full time research years between pre-clinical and clinical years of the medical degree. These MD-PhDs are few, and often organised for individuals rather than part of an established program. Exceptions have been the Universities of Auckland [21] and Sydney [22]. The University of Sydney Medical School’s combined MBBS-PhD program, which enrolled students from 1998 to 2003, reported excellent outcomes in student engagement and productivity [22]. While there are many clinicians with PhDs active in research across Australia, the PhD is commonly undertaken as a medical graduate, after medical school, and often through a specialty training college [17]. Unfortunately, data on their numbers and level of research activity i.e. productivity in grants and publications, are lacking. Traill et al. [17] explored the research activity of medical graduates from one Australian university (1989–2012) and found that undertaking a PhD after medical degree completion took a median time of 13 years. Furthermore, research activity, as evidenced through publications and grants, declined over time. This suggests a lack of opportunity to stay research active perhaps compounded by rising clinical responsibilities in post-graduate training. The reality is that beyond medical school, there are few options to gain a PhD. The primary inadequacy lies in the lack of coordination between funding, protected research time, and clinical commitments to allow young medical scientists to pursue a pathway of integrated research and clinical training [3, 17, 19].

In 2010, the School of Medicine at the University of Queensland (UQ) reflected on the low uptake of medical students into a PhD or Master’s degree. Although the School always offered students the option to do these as intercalated degrees, over a 10-year period (2000–2010), only 13 students had enrolled in either a PhD or Masters. To address this dilemma, the Clinician-Scientist Track (CST) was developed in 2011, representing a model of advanced curriculum for high achieving medical students. It started as a program that allowed enrolment in a part-time research Master’s degree concurrently with the full-time medical degree and was called the ‘Concurrent MBBS-Masters’. The approach was to make the Master’s degree attractive as it was concurrent (i.e. no extra time added to the medical degree), and because a Master’s degree, plus quality research experience would boost students’ career trajectories. The ‘Concurrent MBBS-Masters’, now the Concurrent MD-Masters, represented a two-fold strategy to attract the best students into research and eventually a PhD. In doing so, the importance of clinician-scientist training across the UQ scientific community would be revitalized.

The concept and design of the CST and its early progress 3 years on from its launch has been reported [18]. Eight years since the first enrolments, this article discusses the progress of the CST, lessons learned and recommendations for the future of clinician-scientist training in Australia and similar MD-PhD training schemes elsewhere.

### Structure and timeline for the CST

The medicine program at UQ changed from the MBBS to the MD degree in 2015. The four-year graduate entry degree coincides with the Australian academic year beginning in January, and largely employs a two-semester system. The MD follows the traditional pre-clinical Phase 1, comprising MDY (year) 1 and MDY2 and the clinical Phase 2, comprising MDY3 and MDY4. The change from MBBS to MD did not affect the CST. Students wishing to enter the CST must be eligible to enrol in a Masters or PhD as dictated by the UQ Graduate School. All full-time MD requirements must be met, and a minimum 5.5 grade point average (on a 7-point scale) maintained throughout the MD degree.

Although the CST started with the MD-Concurrent Masters, it offers medical students three RHD options involving a mixture of full- and part-time research activity alongside the MD.

1. *Concurrent MD-Masters* – a research Masters completed on a part-time basis ‘concurrent’ with the full-time MD over MDY2 to MDY4.
2. *Intercalated MD-Masters* – a mixture of full- and part-time research intercalated with the MD. Students interrupt the MD for one full-time research year between MDY2 and MDY3 and return to MDY3 and MDY4 to complete the Masters on a part-time basis.
3. *Intercalated MD-PhD* - a mixture of full- and part-time research intercalated with the MD. Students interrupt the MD for two full-time research years between MDY2 and MDY3 and return to MDY3 and MDY4 to complete the PhD on a part-time basis.

**Fig. 1:**
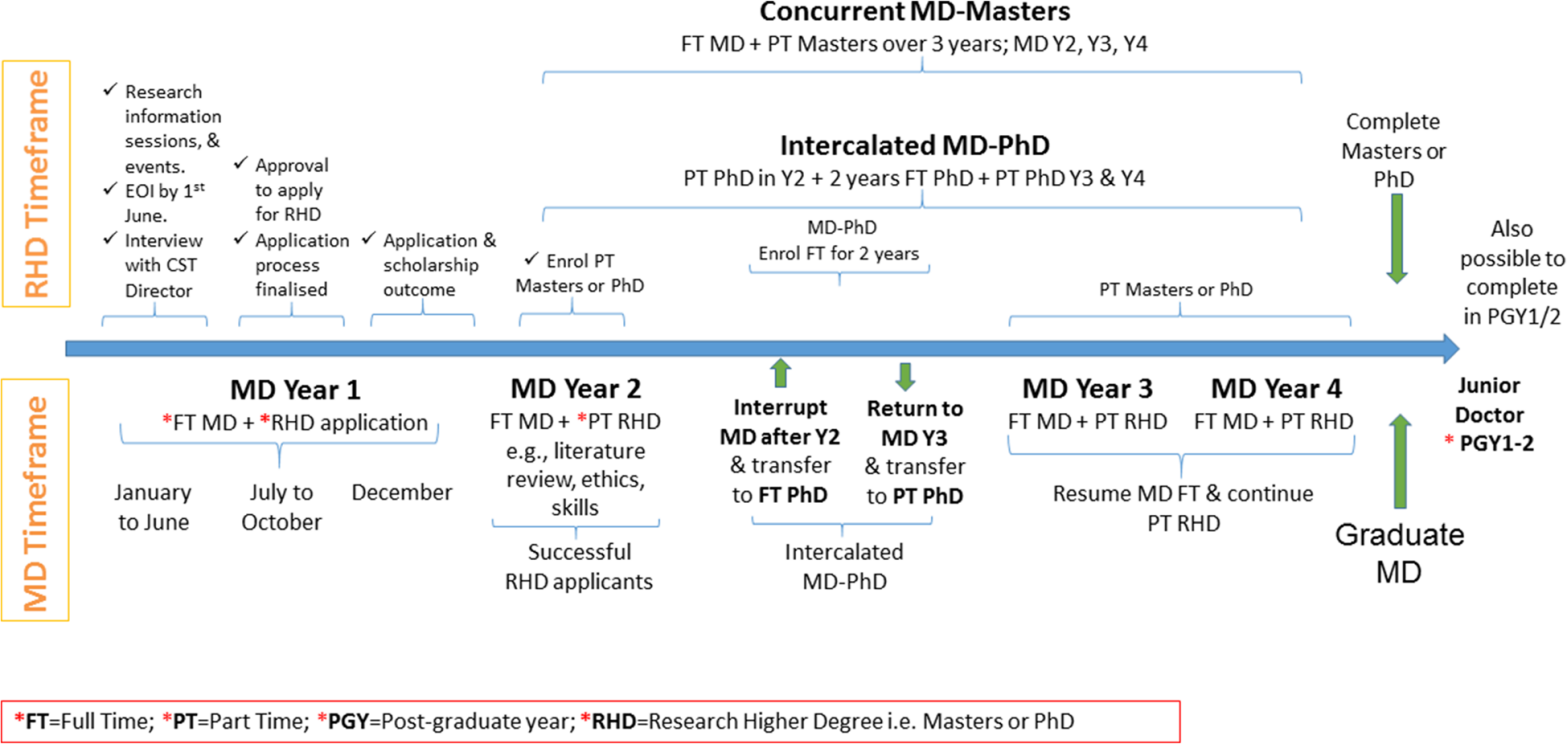
Timeframes for the Clinician-scientist Track

The earliest entry point to the CST is after successful completion of MDY1. This allows students to settle into their MD program, decide if they want to pursue a Masters or a PhD and find a suitable project and a supervisor. The first year MD can be challenging to even the best students and it is important that students’ assess how they will cope with the extra workload and maintain the required grade point. Therefore, entry into the CST involves two approvals. The CST Director must first approve a student to apply for either the Masters or PhD. This involves email correspondence over several weeks or months and at least one face-to-face interview, all of which help ensure that the student and advisory team are committed, capable and understand the program. The second is approval to enrol in the RHD, and the award of a scholarship, which is determined through the normal RHD application process of the UQ Graduate School. Figure 1 outlines the structure and timeframe of the CST.

### Enrolments

**Fig. 2:**
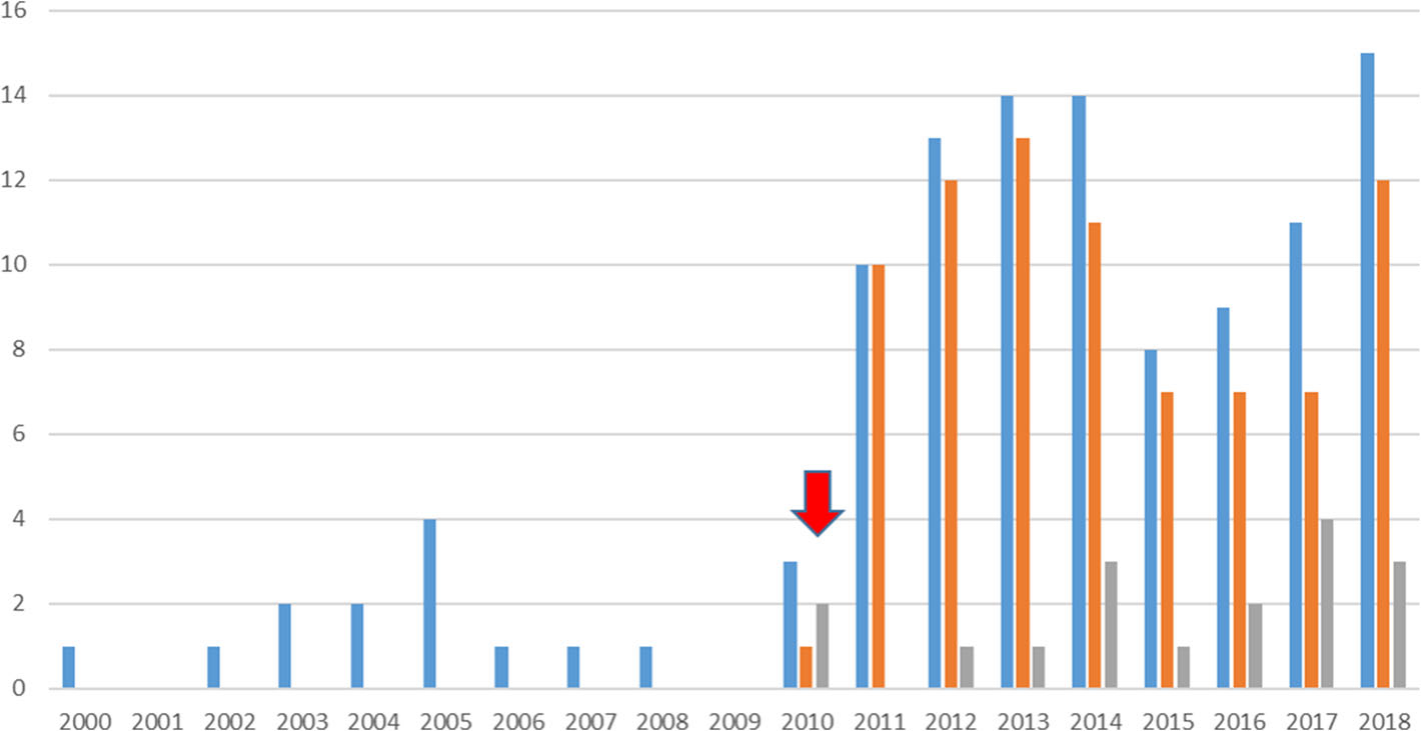
History of medical student enrolments in a Research Higher Degree (Masters or PhD) since 2000. The arrow pointing at 2010 represents the first enrolments in the CST. Blue = Total Research Higher Degree Enrolments, i.e. Masters or PhD. Red = Master’s degree enrolments. Grey = PhD enrolments

In the decade prior to the implementation of the CST, 13 medical students had been enrolled in a RHD. In the eight subsequent years, the numbers of enrolments increased seven-fold to 96. See Fig. 2. Annual enrolments have ranged between 8 and 15. The current number of MD-Masters is 60, (63% of MD-RHD students). The current number of MD-PhDs is 36 (37%). Of all CST enrolments, females represent 39% (*n* = 38). This is equal to the current percentage of females within the whole UQ Medicine Program (40%). The age range (21–26 years), is not different from the whole MD cohort. All but three students entered the MD with a science Bachelor degree.

### Transfers from MD-masters to MD-PhD

The Concurrent MD-Masters was immediately popular and represents the majority of initial RHD enrolments. This initial majority is due in part to RHD eligibility criteria. Often students who intend doing a PhD are required by the Graduate School to start with a Master’s degree and then transfer to the PhD after their first candidature milestone. This is no reflection on the quality of the student, but a rule of the Graduate School. Also to note, as shown in Table 1, the variation in the numbers of transfers from the Masters to the PhD, in particular, enrolling years 2014 and 2016. This discrepancy does not reflect a low up take of the PhD. There is no quota per year for number of students in either the PhD or the Masters, and no requirement for students to transfer to the PhD. This decision is in keeping with our original aim of providing the option to start a Master’s degree as a less daunting goal and provide the option to progress to the PhD. The strategy has proved successful. As shown in Table 1, 19 of the 36 PhDs are transfers from an original Master’s degree, (28% transfers). This figure is encouraging for two reasons: 1) it shows the high level commitment and ability by the student in the additional effort to increase the scope of their project to PhD level, and 2) it supports our goal of training more MD-PhD students and producing more clinician-scientists.

**Table 1:** Record of student numbers and progression in the Clinician-scientist Track

RHD enrolling year	Number of new enrolments	RHD enrolled as:		Transfers from Masters to PhD		International students	UQ Scholarship
		Masters	PhD	Transfer to PhD by ‘n’	Transfer to PhD by ‘%’		
2010^a^	3	1	2	0	0	0	2
2011	10	10	0	5	50%	1	10
2012	13	12	1	3	25%	3	12
2013	14	13	1	4	30%	1	12
2014	14	11	3	1	9%	3	8
2015	8	7	1	3	43%	3	7
2016	9	7	2	1	14%	2	3
2017	11	7	4	2	28%	6	10
2018	14	11	3	0	0^b^	5	14
sub-total	96	79	17	19	28%^b^	24	78
Transfers to PhD		79–19 = 60	17 + 19 = 36				
TOTALS	96	60 (63%) Masters	36 (37%) PhDs		19; 28%^b^	24 (25%)	78 (81%)

Note: ^a^Data from 2010 are included although the official launch of the program was in January 2011

• MD-RHD total enrolments = 96

• MD-Masters = 60; 63% of total MD-RHDs

• MD-PhD = 36; 37% of total MD-RHDs

^b^Transfer of Masters to PhD: 2018 data are not counted in the transfer of Masters to PhD calculations. PhD transfers = 19; 28%. Calculated from 2010 to 2017 which had a total of 68 Master’s enrolments i.e. 19/68 = 28%

• International students = 24 (25%)

• Scholarships awarded = 78 (81%)

### Scholarship support

Support for every CST student is vital to successful progression in both degrees. As noted earlier, with no dedicated pathway of support for MD-PhDs in Australia [17, 19–22], all domestic and international medical students must compete for RHD (Masters and PhD) scholarships with all other university RHD applicants. At UQ, the average number of applicants per year over the period 2013–2017 was PhDs = 2277 and Masters = 516. In Australia, RHD scholarship funding is provided jointly by the Commonwealth Government through the Research Training Program (RTP) [23] and by the UQ’s Graduate School Scholarship. These provide RHD tuition and a living allowance. To date, 81% (*n* = 78) of UQ’s medical students in the CST have been awarded Graduate School Scholarships. Unlike MSTP funding, there is no support for MD tuition or expenses relating to the medical degree. Scholarship funding is sought only for a living allowance and tuition associated with the RHD.

### Research output

Research output from the CST is indicative of success. Over the period 2010–2016, from 71 enrolled MD-RHD students (both Masters and PhD), 159 peer-reviewed papers and 112 conference abstracts were reported (approximately 2.24 papers and 1.58 conference abstracts / CST student). Furthermore all MD-RHD students are first authors on at least one paper. This is comparable with medical student research output reported in similar programs [22, 24, 25]. To note is the inevitable time lag in the peer review and publication process which means that figures of output are always changing, and the process of long term tracking of our graduates can be difficult. We have an online research reporting process for this purpose and encourage its use by all our students and graduates. Publications and journal quality serve as further indicators of research productivity. See Table 2.

**Table 2:** Examples of the quality of student research and productivity. The table shows a sample of some of the more distinguished journals, and their impact factors

Number of publications	Journal	Impact Factor
4	Nature	42.3
2	Lancet	39.2
5	BMJ	16.2
1	Nature Neuroscience	16.1
1	Molecular Psychiatry	14.9
1	Genome Research	14.4
4	Blood	9.8
1	PLOS Genetics	8.5
1	Diabetes	8.5
1	Human Molecular Genetics	7.7
1	Stem Cells	7.1
1	Human Brain Mapping	6.9
1	Circulation: Heart Failure	6.7
3	American Journal of Transplantation	6.2
2	International Journal of Cardiology	6.2
8	Medical Journal of Australia	4.1
5	PLoS One	3.2

### Research areas

Funding for MD-PhDs may understandably impose restrictions on the area and intent of the research pursued by each MD-RHD student. Certainly, funded programs such as the MSTP focus on laboratory-based research congruent with the strengths and priorities of the institution. Nevertheless, there has been increasing encouragement to broaden the scope of clinician-scientist training to include research in the social sciences, public health and health services [26–28]. The value of these research areas and their integral role in the translation of discoveries into patient care is clear. Furthermore, exposing students to research that is relevant and vital to implementing discoveries is what translates to long term advances in health care. Helping students make this connection will not only improve their understanding of research but expand their appreciation of how improvements in health care are implemented.

CST students are encouraged to choose their own research area. During the approval process of a CST applicant, there is equal scrutiny over the integrity of the project and advisory team as on the chosen research area. The world class research centres and institutes at UQ is intense and diverse and therefore ensures CST students undertake projects that are focussed on national and regional priorities. Furthermore, encouraging candidates to choose their own project enhances motivation and allows them to align their research interests with their clinical career plans. This may further enhance the meaningful connections between their research findings and clinical practice. Among the CST cohort to date, 45% of projects are within clinical research, 37% are biomedical wet lab research, and 16% of students have chosen clinically relevant projects within epidemiology, health services, and environmental studies. The only restriction imposed on project type is that students choosing the Concurrent MD-Masters must undertake research that is flexible with no time critical elements i.e. no wet-lab work. This is to help ensure it can be accommodated alongside the full-time MD degree. Ideal projects for the concurrent MD-Masters include epidemiology, biostatistics, health services and public health research. In contrast, wet-lab/basic science and clinical research are encouraged for the intercalated MD-PhD and intercalated MD-Masters because they include dedicated research years during which time-critical elements and the bulk of data are collected.

### Program progression

The number of RHD completions is perhaps the most important measure of the CST’s progression. An accurate RHD completion figure takes into account the protracted timeframe for MD-Masters and MD-PhD, and the full-time/part-time model for each. While this tends to extend the overall completion time, it does not exceed the full-time equivalent limits for the Masters or PhD as dictated by the UQ Graduate School. See Fig. 1. For example, of the 54 enrolments over the 2010–2014 period, 25 RHDs were completed (17 Masters, 8 PhDs) giving 46% completion thus far.

An equally important indicator of the program is the number of withdrawals. To date 14 (14%) students have withdrawn. This figure over 8 years is within the range of what has been reported in the literature [29–31]. The average attrition of MD-PhD programs in the US was between 10 and 29% [4, 29, 30], with a Swiss study reporting 8% [31]. The reasons for CST withdrawals are varied. Five were early enrolments in the program when there were several unknowns, such as the level of commitment and extra workload required, as well as understanding the best combination of student and supervisor characteristics most conducive to this model. The main reasons are related to the inflexible early post-graduate intern years allowing little or no time protected time to finalise the thesis if necessary after MD graduation.

### Challenges and solutions

A challenge of the CST reflects the lack of integration in administration and progression of the largely separate degrees. This is most obvious in that students do not matriculate as an MD-PhD, or MD-Masters student, often termed a ‘dual-degree’. As outlined in Fig. 1, during MDY1, or MDY2, students first request Faculty approval to then apply for Graduate School approval to enrol in the PhD or Masters and be considered for a scholarship. While there are clear stages to each process, confusion can arise between the timelines for each. Furthermore, there is no requirement for students to complete the RHD before or coincident with MD graduation. Consequently, completing both degrees while in the medical program i.e. over four (Masters) or six (PhD) years is not the norm. Most students complete the RHD during intern (early post-graduate) years but there is pressure to stay within the maximum time allowed for the RHD as determined by the UQ Graduate School. Creation of a dual-degree would help address both these issues; by streamlining the process of combining the RHD with the MD, and providing support for students in the hope of completing both degrees prior to post-graduate training. This dual-degree model would elevate recognition of students in the MD-Masters and MD-PhD as a distinct cohort and further raise awareness of and help shift the culture in Australia toward specific clinician-scientist training during medical school.

Another challenge is to keep undergraduate pre-medical students informed of their research options prior to entering the medical program. The CST program engages the undergraduate UQ Pre-Medical Society to raise awareness of the research opportunities in the MD program and to help identify outstanding students with clear goals for combining a clinical and research career. Information, meet-and-greet evenings, and research Expos, include the entire UQ research community where potential supervisors present their research to pre-med Bachelor’s students in hopes of attracting prospective MD-PhDs. The rich UQ research community is a strong supporter, indeed a partner, of the CST and vital to its success. Again, offering matriculation in a dual-degree would provide the initial stage of a clinician-scientist pathway, where students may aspire to a clear goal which should ideally begin during the Bachelor’s degree.

A challenge for most MD-PhD programs is how to lessen the effect of sudden transitions from research to clinical environments over the course of the MD-PhD where students interrupt the MD for full-time research. Recent literature has used the metaphor of the clinician-scientist as having ‘feet in two boats’ [32], which is also applicable to MD-RHD training. More flexibility in the MD curriculum and within post-graduate years would enhance the ethos of integration in clinician-scientist training. While the pipeline analogy works well as a long and leaky journey, it assumes inevitable leaking somewhere sometime. Perhaps more appropriate and beneficial to learning, is the notion of multiple research ‘on-ramps’ throughout this journey [33]. Hall [33] describes this model as appropriate anywhere along the pipeline as a means of accessing research opportunities that integrate with clinical phases rather than abrupt transitions from intense research to intense clinical training. This, more flexible, integrative model would serve to enhance students’ appreciation of the clinical relevance of their research, and likewise, the research that underlies their clinical training. Furthermore, this integration may ease the stressful transitions reported by students and junior doctors due to their abrupt nature, unclear expectations and feelings of unpreparedness [34, 35].

A future CST solution provides the integration of clinical exposure and enhancement of skills within the research years of the MD-PhD. Clinical skills sessions for RHD students are administered at the end of each academic year comprising half and/or day long ‘intensives’ of clinical skills. This refresher program of clinical skills helps ensure students maintain clinical focus, while importantly allowing the majority of time devoted to research. The program aims to further develop and maintain a clinical learning environment by providing clinical contact across the years of research and help ease the transition back in to the MD.

## Conclusions

This paper has discussed the progression of a program of clinician-scientist training in Australia, now in its eighth year. The philosophy behind the CST from the outset was congruent with Ley and Rosenberg [36] positing the notion of “build it, and they will come”. The CST began with a concurrent MD-Masters to attract medical students to MD-PhDs and this strategy is demonstrating early signs of success. It is too early to comment on the career trajectory of CST alumni. Those graduates who are at least 4 years into postgraduate training, and have managed to maintain close contact with the supportive environment of their PhD training, remain research active, achieving early career researcher awards, grants, and qualifying for academic titles. However the early post-graduate training years can mean relocation to hospitals with little or no support to continue research. This situation, as noted by Windsor et al. [2], is an example of the shared responsibility that is necessary between institutions and stakeholders to support and nurture our newly trained clinician-scientists. The most immediate career advantage for CST graduates is gaining competitive speciality and fellowship training positions.

Will they stay on track to become scientists or will they focus on clinical work? Australian literature suggests that lack of support for dedicated research time during postgraduate training, as an intern, junior doctor and registrar, is a significant challenge [16, 17, 19, 20]. Early studies report considerable challenges to MD-PhD graduates in following a clinician-scientist career and high attrition among females [37]. In contrast a recent US study tracked MD-PhD graduates over 50 years and found 80% of respondents are following career paths consistent with the goals of clinician-scientist training with 50% spending half their time conducting research alongside clinical activities [8, 38]. However, the study also notes that despite females making up half of all students in US medical schools, only 35% make up MD-PhD places. At 39%, the proportion of females in the CST is reflective of the UQ MD cohort, but this itself is considered low. The CST at present does not specifically target female students and enrolments have fluctuated between 20 and 60% each year. Certainly there is no difference in their progression and performance in the CST. The literature continues to note the challenge of this gap between male and females in MD-PhD training [37–39]. Furthermore, while there are no differences in choices made regarding workplace after graduation, there continues to be large differences in salaries awarded to males versus females in research and academic positions [39].

Our 8 year experience with the CST, albeit a model in one university, provides recommendations for the future of the program, and similar MD-PhD schemes elsewhere. Foremost among these recommendations is a coordinated approach to a supported program – an Australian pathway – for our most talented and committed students beginning in the undergraduate Bachelor degree, into the MD degree and throughout speciality training. The greatest barrier to students, considering an MD-PhD is the gap to a clear career pathway for training [20, 40]. Coordinated institutional collaboration between university medical schools, state health systems and speciality colleges might offer multiple research ‘on-ramps’ providing both integration and continuity in training. Flexibility at all points along this training continuum is required, and importantly to acknowledge the lifestyle needs of an emerging generation of clinician-scientists who have varied expectations for their careers. This is especially true for females who are too often the victims of outdated inflexible programs, at odds with family plans or obligations [37]. Without addressing this gap to a pathway, we risk losing the next generation of potential clinician-scientists who are otherwise aware of, and eager to take on the challenges of the MD-PhD journey, but understandably expect to receive appropriate support along the way. The required ‘commitment’ to help establish a training pathway should be considered a necessary ‘investment’ in our future clinician-scientist workforce.

## Abbreviations

Masters: Master of Science research higher degree
MD: Medical doctor degree
MD-Masters: Dual degree program combining an MD and a Masters
MD-PhD: Dual degree program combining an MD and PhD
MPhil: Masters of Philosophy research higher degree which is a common term in Australia and Europe for the Masters of Science degree. They are equivalent research higher degrees
PhD: Doctor of Philosophy research higher degree
RHD: Research Higher Degree comprising the PhD and Master of Science
